# Global temporal dynamic landscape of pathogen-mediated subversion of Arabidopsis innate immunity

**DOI:** 10.1038/s41598-017-08073-z

**Published:** 2017-08-10

**Authors:** Bharat Mishra, Yali Sun, Hadia Ahmed, Xiaoyu Liu, M. Shahid Mukhtar

**Affiliations:** 1 Department of Biology, University of Alabama at Birmingham, Birmingham, USA; 2 Department of Computer & Information Sciences, University of Alabama at Birmingham, Birmingham, USA; 3 Nutrition Obesity Research Center, University of Alabama at Birmingham, Birmingham, USA

## Abstract

The universal nature of networks’ structural and physical properties across diverse systems offers a better prospect to elucidate the interplay between a system and its environment. In the last decade, several large-scale transcriptome and interactome studies were conducted to understand the complex and dynamic nature of interactions between Arabidopsis and its bacterial pathogen, *Pseudomonas syringae* pv. tomato DC3000. We took advantage of these publicly available datasets and performed “-omics”-based integrative, and network topology analyses to decipher the transcriptional and protein-protein interaction activities of effector targets. We demonstrated that effector targets exhibit shorter distance to differentially expressed genes (DEGs) and possess increased information centrality. Intriguingly, effector targets are differentially expressed in a sequential manner and make for 1% of the total DEGs at any time point of infection with virulent or defense-inducing DC3000 strains. We revealed that DC3000 significantly alters the expression levels of 71% effector targets and their downstream physical interacting proteins in Arabidopsis interactome. Our integrative “-omics”-–based analyses identified dynamic complexes associated with MTI and disease susceptibility. Finally, we discovered five novel plant defense players using a systems biology-fueled top-to-bottom approach and demonstrated immune-related functions for them, further validating the power and resolution of our network analyses.

## Introduction

While plants are exposed to a wide range of biotic and abiotic stresses, they possess a robust and resilient cellular architecture that allows them to adapt to constantly changing environments^1, 2^. As a result, the plant cells display a remarkable capacity to maintain their homeostasis. This is achieved through distinct genes and their products, which function in concert in response to diverse environmental cues^3^. These cellular products including nucleic acids, proteins, hormones, ions, metabolites or other macromolecules are highly interactive with each other, making it pertinent to understand cellular functions in a complex system^1^. Generally, a biological system constitutes a network of interconnected cellular components. The structural, physical, and functional connections among these components are termed edges, while the molecules involved in the interactions are referred to as nodes^4^. The widespread utilization of genome-wide or “-omics” approaches that seek to integrate large-scale data such as genomes, proteomes, transcriptomes, interactomes, and others into descriptive models, helps elucidate the molecular functions at the systems level^2, 3, 5^. Indeed, such an integrative platform has been recently proposed to decipher the underlying molecular mechanisms of plant disease resistance, in particular the pathogenesis of a deadly necrotrophic fungus *Botrytis cinerea* ^5^. Moreover, network biology and other computational as well as integrative tools play an increasingly essential role in decoding the genetic information between the genome and the plant phenome that comprises multiple levels of information processing *via* different “-omes”^6–10^. Thus, network biology is rooted in discovering biological relationships between different cellular components and environment as well as providing deep insights into inter- and intracellular communication processes^10–12^.

The plant–microbe pathosystem constitutes a very complex biological network, in which the molecular players from both pathogens and their hosts engage in a battle for dominance^13^. Extracellular microbes including pathogenic bacteria enter plant tissue through natural openings such as stomata and reside in the extracellular spaces known as the apoplast^14^. To detect the danger signals as well as invading pathogens, plants have evolved a repertoire of pattern recognition receptors (PRRs) localized to the plasma membrane. Receptor-like kinases (RLKs), a sub-group of PRRs that display structural and functional similarities to the extracellular leucine rich repeat (LRR) domain of mammalian Toll-like receptors (TLRs), in interactions with other receptors, perceive conserved microbe-associated molecular patterns (MAMPs)^15^. This receptor-ligand direct interaction leads to a series of cellular events and triggers effective immune responses known as MAMPs-triggered immunity (MTI)^15, 16^. Highly studied examples of MAMPs include bacterial flagellin and elongation factor Tu (EF-Tu) that are recognized by FLS2 (Flagellin Sensitive 2) and EFR (EF-Tu Receptor) RLKs, respectively^15^. In addition to PRRs, plants have also evolved another class of sensors known as nucleotide binding-LRR (NLR) receptors. These NLRs can directly perceive pathogen virulence molecules (hereafter called effectors) or monitor the activities of effectors on host proteins (indirect recognition) and initiate a defense response termed effector-triggered immunity (ETI)^17–21^. ETI is characterized by quantitative defense responses ranging from heightened immunity to hypersensitive response (HR), similar to programmed cell death (PCD). Both MTI and ETI are manifested by the enhanced generation of reactive oxygen species (ROS), stimulation of downstream mitogen-activated protein kinases (MAPKs), release of antimicrobial peptides in the apoplast and interplay of hormonal crosstalk^22–24^. Additionally, the activation of these defenses also involves massive spatiotemporal transcriptional reprogramming involving intricate signal transduction pathways through largely unknown mechanisms^22, 24, 25^. Meanwhile, specialized pathogens such as the bacterial pathogen *Pseudomonas syringae* secrete and deliver effectors into the plant cells^26^ using type three secretion system (TTSS). These effectors interfere with MTI and/or ETI at various levels and establish effector-triggered susceptibility (ETS)^19, 27^. Likewise, effector molecules manipulate phytohormones-mediated transcriptional crosstalk for their advantage and facilitate ETS^13^; however, the underlying molecular mechanisms are not fully understood.

In the last decade, several large-scale genome-wide expression studies have been designed to identify the functions of the pathogen effectors^28^. Very recently, a high-resolution temporal transcriptome analysis was performed in Arabidopsis using a virulent bacterial pathogen DC3000 that triggers ETS^29^. Additionally, an attenuated strain *P. syringae* pv. tomato DC3000*hrpA* ^−^ (hereafter DC3000*hrpA* ^−^ *)* was also included. DC3000*hrpA* ^−^ possesses the whole suite of MAMPs but lacks a key structural component of the TTSS pilus and hence cannot deliver effector molecules into the plant cell. Thus, DC3000*hrpA* ^−^ can effectively activate MTI but is incapable of establishing ETS^29^. This elegant study conducted by Lewis *et al*.^29^ revealed how virulent DC3000 dynamically reshapes the global transcriptional landscape to establish ETS. In addition to transcriptome studies, several global protein–protein interaction (PPI) networks (interactomes) were also generated for Arabidopsis^30–36^. Specifically, we constructed Arabidopsis Interactome version 1 “main screen” (AI-1_MAIN_) using ~8,000 Arabidopsis open reading frames. AI-1_MAIN_ contains 5,664 interactions between 2,661 proteins^32^ and was generated by the “Arabidopsis Interactome Mapping Consortium”. Furthermore, we also generated two Plant-Pathogen Interaction Networks, *i.e*. PPIN-1 and PPIN-2 featuring interactions among proteins from the bacterium *P. syringae*, an oomycete *Hyaloperonospora arabidopsidis* (HPA), a fungus *Golovinomyces orontii* (GOR) and host Arabidopsis^30, 33^. PPIN-1 was a collaborative project among several individual laboratories and the European Union Effectoromics Consortium^33^. Overall, these interactome maps revealed that effectors from diverse pathogens target 201 host proteins (effector targets). To understand the dynamic transcriptional and protein-protein interaction behaviors of effector targets, we took advantage of high-resolution dynamic transcriptome data generated by Lewis *et al*.^29^ as well as interactome data extracted from AI-1_MAIN_, PPIN-1, and PPIN-2, and performed additional network biology analyses.

Network analyses discovered that these effector targets possess a high degree (number of connections) and uncovered that evolutionarily diverged pathogens independently evolved effectors to converge onto a limited set of shared effector targets^30, 32, 33^. While these large-scale PPI networks and network biology analyses provided profound insights into the topological features of the effector targets, such interactomes only exhibit a static view of the cellular organization^37^. Given that biological systems are highly dynamic and proteins play multifunctional roles in spatiotemporal micro-environments, dynamic modeling by integrating diverse “-omics” data represents a new frontier in network biology. Such network analyses, in conjunction with diverse computational tools and predictive modeling, elucidate the spatiotemporal organization of the molecules and their interactions under diverse physiological cellular states. Here, we demonstrated that effector targets exhibit increased information centrality. The shortest distance between effector targets and differentially expressed genes (DEGs) is lower than that of non effector targets and DEGs. Our data suggest that approximately 1% of the effector targets are differentially expressed at any given time of the transcriptional responses during MTI or ETS. We also showed that over 70% of the MTI-related effector targets and their downstream physical interacting partners are differentially regulated by ETS. Network analyses of the unique ETS-response effector targets and their downstream interactors may lend deeper insights into how the virulent bacterial pathogen DC3000 suppresses MTI and other defense-related processes for their benefits. Finally, our dynamic subnetwork analysis identified novel five key proteins involved in plant immunity.

## Results

### Integration of transcriptome to interactome reveals that effector targets are closer to differentially expressed genes (DEGs)

**Figure 1:**
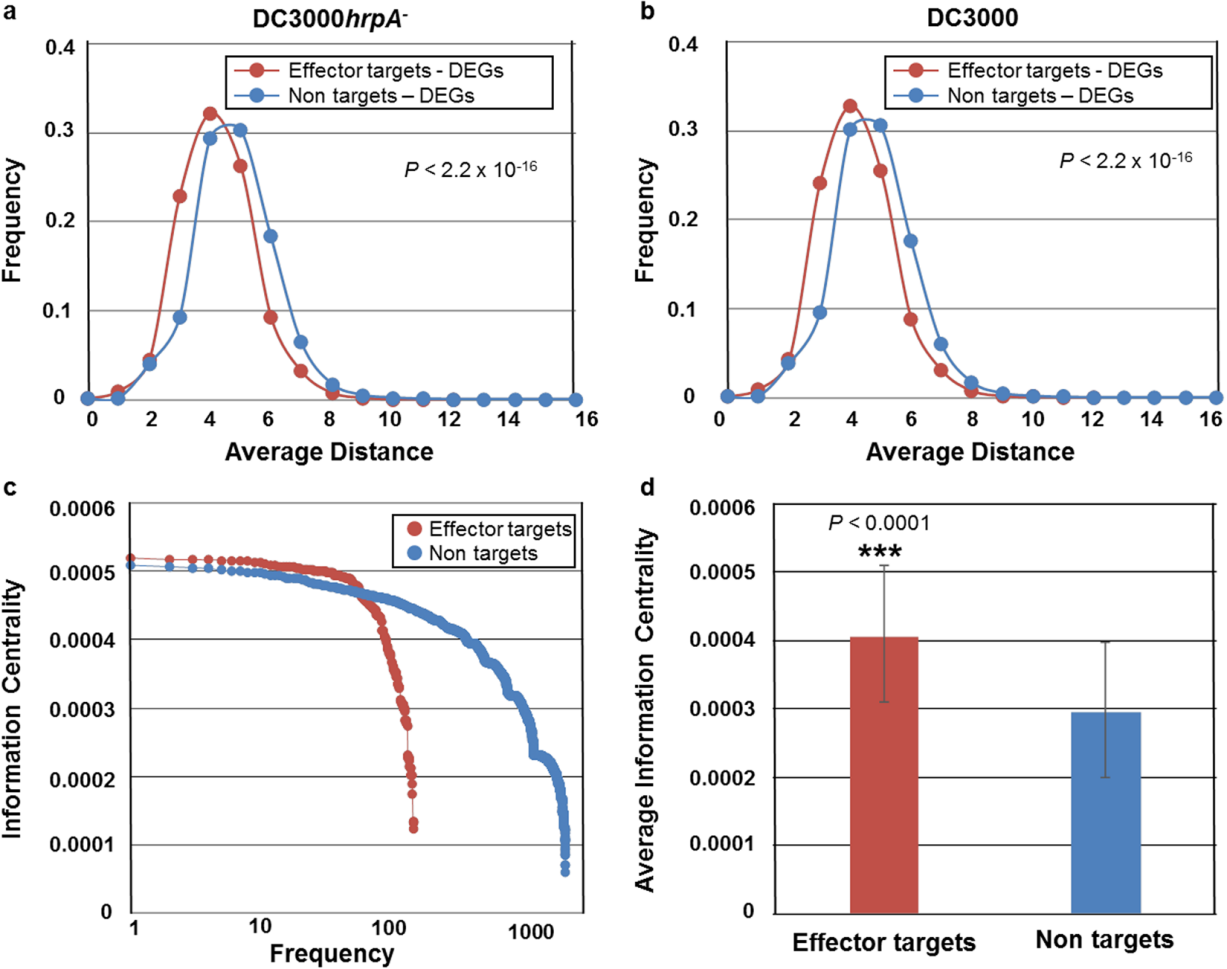
Measurement of diverse centrality parameters for effector targets. (**a** and **b**) Distribution of shortest path for pairs of effector targets and differentially expressed genes (DEGs) (magenta), and non effector targets and DEGs pairs (blue) for DC3000*hrpA* ^−^ (**a**) and DC3000 (**b**). (Chi-Square Test of Independence; *P* < 2.2 × 10^−16^). (**c**) Distribution of information centrality for effector targets (magenta) and non targets (blue) in AI-_MAIN_. Frequency of occurrences of information centrality for both categories are depicted. (Student’s *t*-test *P* < 0.0001). (**d**) Average information centrality for effector targets (magenta) and non targets (blue) in AI-MAIN is displayed. (Student’s *t*-test *P* < 0.0001).

A wide range of multifaceted systems including social, biological, and technological networks display hierarchical organizations and exhibit universal topological features^6, 38, 39^. In these diverse systems, different centrality measures^40^ including degree (number of links of a node), shortest paths (shortest distance between two nodes), and betweenness (number of shortest paths pass through a node), etc., might determine the transmission of information between two nodes in a connected network^41^. Given that effector targets exhibit an increased degree compared to non targets^31–33^, we hypothesized that diverse pathogens interact with effector targets to interfere with the flow of information. Global gene expression constitutes another central parameter required for the transmission of biological information. Thus, we integrated a high-resolution pathogen-related transcriptome^29^ into AI-1_MAIN_^32^. This genome-wide transcriptional study identified 7,119 and 9,782 differentially expressed genes (DEGs) for DC3000*hrpA* ^−^ and DC3000, respectively and 11,070 cumulative DEGs for any of these two treatments. This transcriptome study provided a timeline for the onset of MTI and immunosuppression of host defense triggered by DC3000. Here, we focused our analyses to specifically investigate the dynamic relationships between effector targets and non targets and DEGs induced by either DC3000*hrpA* ^−^ or DC3000. To examine the flow of information between two given nodes in a network, we measured the shortest distance between effector targets and DEGs associated with DC3000*hrpA* ^−^, DC3000 or any treatment. We demonstrated that the distance between effector targets and DEGs are significantly shorter than non targets and DEGs, irrespective of any treatment (Chi-Square Test of Independence; *P* < 2.2 × 10^−16^, Fig. 1a,b; Supplementary Fig. S1). In addition to the shortest path, information centrality that measures the transmission of information between any two points in a connected network is another feature that can describe the flow of information in a network^41, 42^. While information centrality is initially described in the propagation of information in social networks^42^, it can be adapted for biological networks to study the most influential information spreader nodes in the network^43^. Thus, we calculated the information centrality for effector targets and non targets in AI-1_MAIN_. The information centrality distribution of effector targets reveals that the effector targets have a significantly higher information centrality compared to non targets in AI-1_MAIN_ (*P* < 0.0001) (Fig. 1c). Furthermore, we also calculated average information centrality for effector targets and non targets in the largest connected component of AI-1_MAIN_. As shown in Fig. 1d, the average information centrality of 0.0004 for effector targets is significantly greater than that of non targets (0.0003; *P* < 0.0001). These data corroborate with previous findings in diverse host-pathogens systems including human-*Burkholderia mallei* network^44, 45^. Collectively, our data suggest that Arabidopsis relies on effector targets to spread information through DEGs, and pathogen effectors may rewire the flow of information by interacting with these high value targets.

### Sequential and temporal regulation of effector targets in innate immunity and disease susceptibility

**Figure 2:**
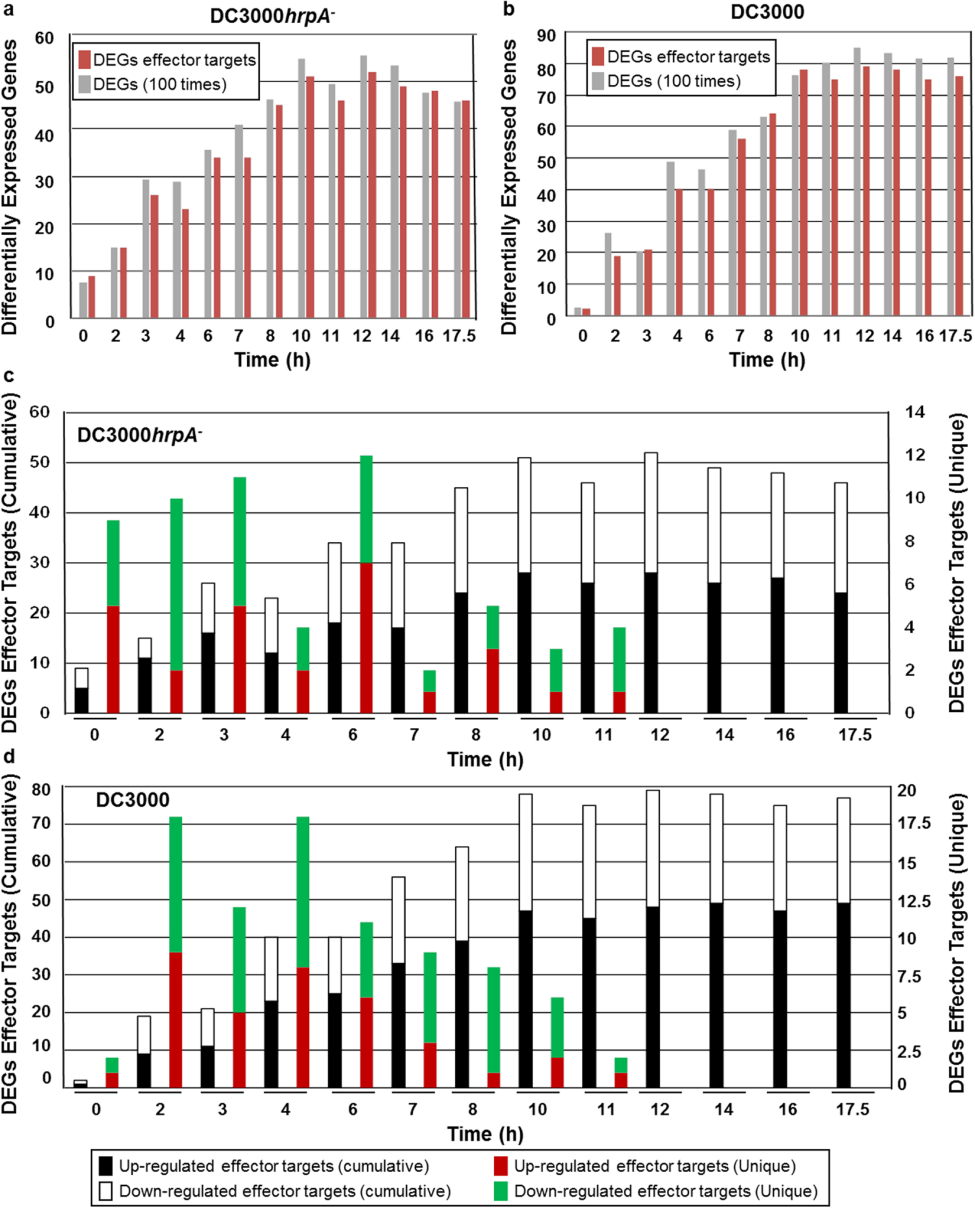
Temporal regulation of effector targets. (**a** and **b**) The histograms illustrate total number of differentially expressed genes (DEGs, grey) and effector targets that are differentially expressed (magenta) at the indicated time points for DC3000*hrpA* ^−^ (**a**) and DC3000 (**b**). Note that the value of total DEGs are recorded 100 times more than DEGs effector targets. (**c** and **d**) The histograms of cumulative DEG effectors and first gradient change in transcription for effector targets (DEGs effector target unique) are displayed at the indicated time points for DC3000*hrpA* ^−^ (**c**) and DC3000 (**d**). Up- and down-regulation of genes are shown in black (DEGs effector target cumulative) and red (DEGs effector target unique), white (DEGs effector target cumulative) and green (DEGs effector target unique), respectively.

**Figure 3:**
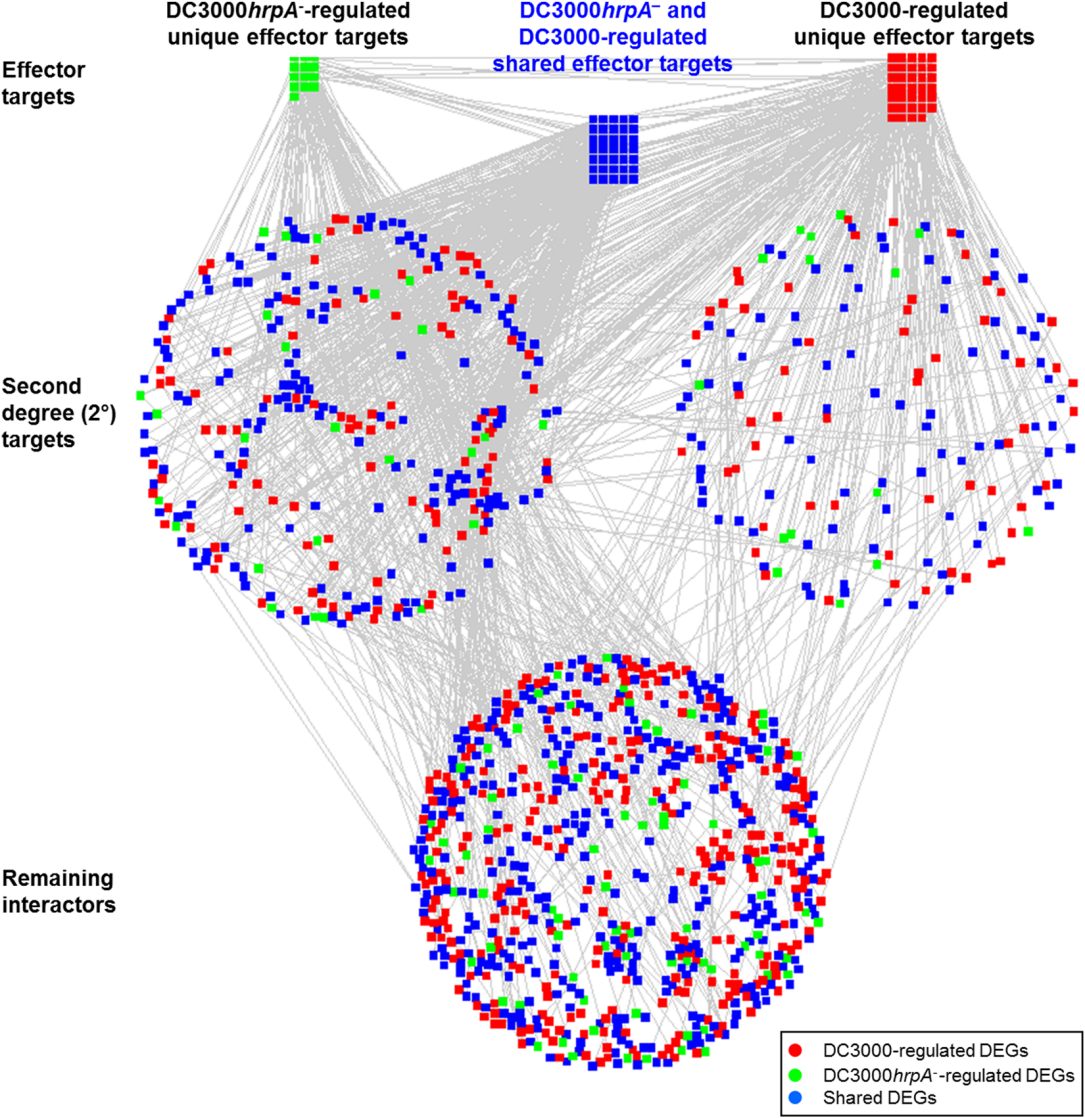
Visualization of DC3000*hrpA* ^−^ and DC3000-regulated effector targets. Unique and shared effector targets that are differentially expressed by DC3000*hrpA* ^−^ and DC3000 in AI-_MAIN_ are illustrated in the first layer of the network. Second degree targets and remaining proteins make second and third layers in AI-_MAIN_, respectively. Non DEGs are removed from AI-_MAIN_ for clarity.

To better understand the transcriptional and protein-protein interaction activities of effector targets, we determined the temporal regulation patterns of the genes encoding effector targets in a fine time-course genome-wide expression data set generated by Lewis *et al*.^29^. This study identified DC3000*hrpA* ^−^- and DC3000-regulated genes and revealed expression gradient changes in DEGs at a given time point. In our study, we centered our analysis on effector targets in the context of total DEGs regulated by DC3000*hrpA* ^−^ and DC3000. Remarkably, we discovered that 56% (113 out of 201) of the effector targets are differentially expressed by both DC3000*hrpA* ^−^ and DC3000 treatments (Supplementary Table S1). Overall, we found that effector targets equate to 1% of the total DEGs that are differentially regulated by DC3000*hrpA* ^−^ and DC3000 treatments, respectively (Supplementary Table S1). Next, we displayed the total number of DEGs and DEGs effector targets over the course of 17.5 hours (h) treatments with DC3000*hrpA* ^−^ and DC3000. Importantly, we again revealed that approximately 1% of the effector targets are differentially expressed in the total number of DEGs at any time point irrespective of the type of bacterial strain used for the infection (Fig. 2a, b). First significant divergence in the expression gradient analysis revealed that a substantial number of unique effector target genes are differentially expressed upon infection with DC3000*hrpA* ^−^ at early time points, peaking at 6 h (Fig. 2c; Supplementary Table S2). Unlike DC3000*hrpA* ^−^, however, we observed that down-regulated group of effector targets, which are diverged in their expression gradients for the first time, are more prevalent than their up-regulated counterpart group between 7 h and 11 h time points after infection with DC3000 (Fig. 2d; Supplementary Table S2). Overall, we demonstrated a sequential and temporal regulation of effector targets over the course of infection with DC3000*hrpA* ^−^ and DC3000. To decipher the dynamic relationships of effector targets between innate immunity and disease susceptibility, we extracted MTI- and ETS-regulated 63 and 86 effector targets, respectively in AI-1_MAIN_ (Supplementary Table S3). We found that expression patterns of 71.45% (45 out of 63) of the MTI-regulated effector targets are significantly altered by DC3000 (Fig. 3, Supplementary Fig. S2 and Supplementary Table S3). Moreover, we also identified a set of proteins that physically interact with effector targets in AI-1_MAIN_^32^. We termed these interacting partners of an effector target as “second degree (2°) targets” (Supplementary Table S3). This analysis discovered that over 78% of 2° targets are shared between MTI- and ETS-regulated genes (Supplementary Table S3). Finally, we visualized temporal interactome dynamics in MTI and ETS over the course of 17.5 h post infection (Supplementary movies S1 and S2). These data revealed the dynamic regulation of DC3000*hrpA* ^−^ controlled effector targets and their downstream physical interacting partners. Given the high connectivity of effector targets in AI-1_MAIN_^32^ and increased information centrality (Fig. 1), these data also suggest that host likely transmits MTI-related information *via* these high centrality first degree and 2° effector targets. Moreover, DC3000 alters the expression patterns of these effector targets and interferes with the flow of information to establish disease.

### Identification of temporal protein complexes in plant immune system

While the technological advances allow the construction of large-scale PPI networks using several high throughput methods such as Y2H, such interactomes, however, merely illustrate a snapshot of interactions among proteins. Given that proteins may establish both stable and dynamic connections under diverse physiological conditions, elucidating how protein complexes are organized in a network will illuminate the significance of certain nodes that may possess influential topologies^37^. To identify temporal protein complexes, we integrated DC3000*hrpA* ^−^- and DC3000-related time-course datasets into AI-1_MAIN_. This comprehensive analysis fostered the discovery of MTI- and ETS-specific dynamic protein complexes (Supplementary Table S4), which can form the basis for future investigations. To infer their roles in plant immune system, we selected five such dynamic complexes comprising genes that display divergent expression patterns during the late phase of infection with DC3000 and/or DC3000*hrpA* ^−^ (Supplementary Table S5). The first co-regulated dynamic complex contains At2g04030 (AtHSP90.5; heat-shock protein90.5 also known as CR88) and a PAS domain-containing protein tyrosine kinase family protein (At4g23050; MAP3K*δ*4, a member of the RAF-like mitogen-activated protein 3 kinases; MAP3Ks)^46^ (Supplementary Table S5). CR88 is a molecular chaperone that is involved in sensitivity to diverse oxidative and abiotic stresses including salt and drought^47^. Since plant hormone abscisic acid (ABA) is required for plant responses to drought as well as involved in stomatal-induced plant immunity^48^, it is likely that CR88 may participate in MTI. Intriguingly, the transcript levels of *MAP3Kδ4* are also induced by ABA, salt, drought and cold conditions^46^. Moreover, overexpression of *MAP3Kδ4* was shown to confer salt tolerance in Arabidopsis^46^ suggesting that *MAP3Kδ4* might be involved in plant defense, possibly through ABA-mediated crosstalk with plant defense-responsive phytohormone such as salicylic acid (SA). The second dynamic complex is composed of JAZ5 (Jasmonate ZIM domain containing transcriptional repressor; At1g17380) and NINJA (Novel Interactor of JAZ; At4g28910) (Supplementary Table S5). The JAZ proteins form repressor complexes by recruiting NINJA and several other corepressors, and modulate gene expression-mediated by jasmonic acid (JA) signaling pathway^49, 50^. The functions of JAZ5 in JA- and SA-mediated crosstalk as well as plant immunity have been very well characterized^51^. In our analysis, SQD1 (sulfoquinovosyl diacylglycerol 1; At4g33030) forms third dynamic complex with At5g56350, a putative pyruvate kinase family member (Supplementary Table S5). SQD1 is a UDP-sulfoquinovose-synthase that converts UDP-glucose into UDP-6-sulfoquinovose^52^ and is involved in the biosynthesis of sulfolipids in Arabidopsis^53^. Whereas, pyruvate kinase catalyzes the final step in glycolysis and produces acetyl-CoA^54^, a central molecule to a wide range of biochemical reactions including degenerative pathways and oxidative-fueled metabolism^55^. While the roles of primary metabolism in plant defense and pathogen infection are emerging^56, 57^, the potential functions of SQD1 and pyruvate kinase in plant-pathogen interactions have not been fully explored. The fourth dynamic complex encompasses AFG1-like ATPase (ATPase family gene 1; At4g30490)^58^ and At5g61010 (EXO70E2, an exocyst subunit) (Supplementary Table S5). Importantly, several members of AAA^+^-ATPase superfamily as well as EXO70 subunits have been implicated in plant defense^59–61^. However, the plant immune functions of these specific members of ATPase and EXO70 families are yet to be determined. Likewise, plant defense-related activities remain to be discovered for the fifth dynamic complex that is composed of At1g06460 (ACD31.1; alpha-crystallin domain31.1^62^), At2g24860 (DnaJ/Hsp40 cysteine-rich domain superfamily protein) and At4g35250 (HCF244, a member of the atypical short-chain dehydrogenase/reductase superfamily, a modified group that has lost enzyme activity) (Supplementary Table S5). To further our understanding of the potential functions of these dynamic complexes in the plant immune system, we investigated the roles of five genes, one from each dynamic complexes. These include DC3000-specific CR88, JAZ5 and SQD1, DC3000*hrpA* ^−^- specific AFG1-like ATPase as well as DC3000*hrpA* ^−^- and DC3000-regulated ACD31.1^62^ (Supplementary Table S5).

### Isolation of dynamic subnetworks identifies novel players in plant disease resistance

**Figure 4:**
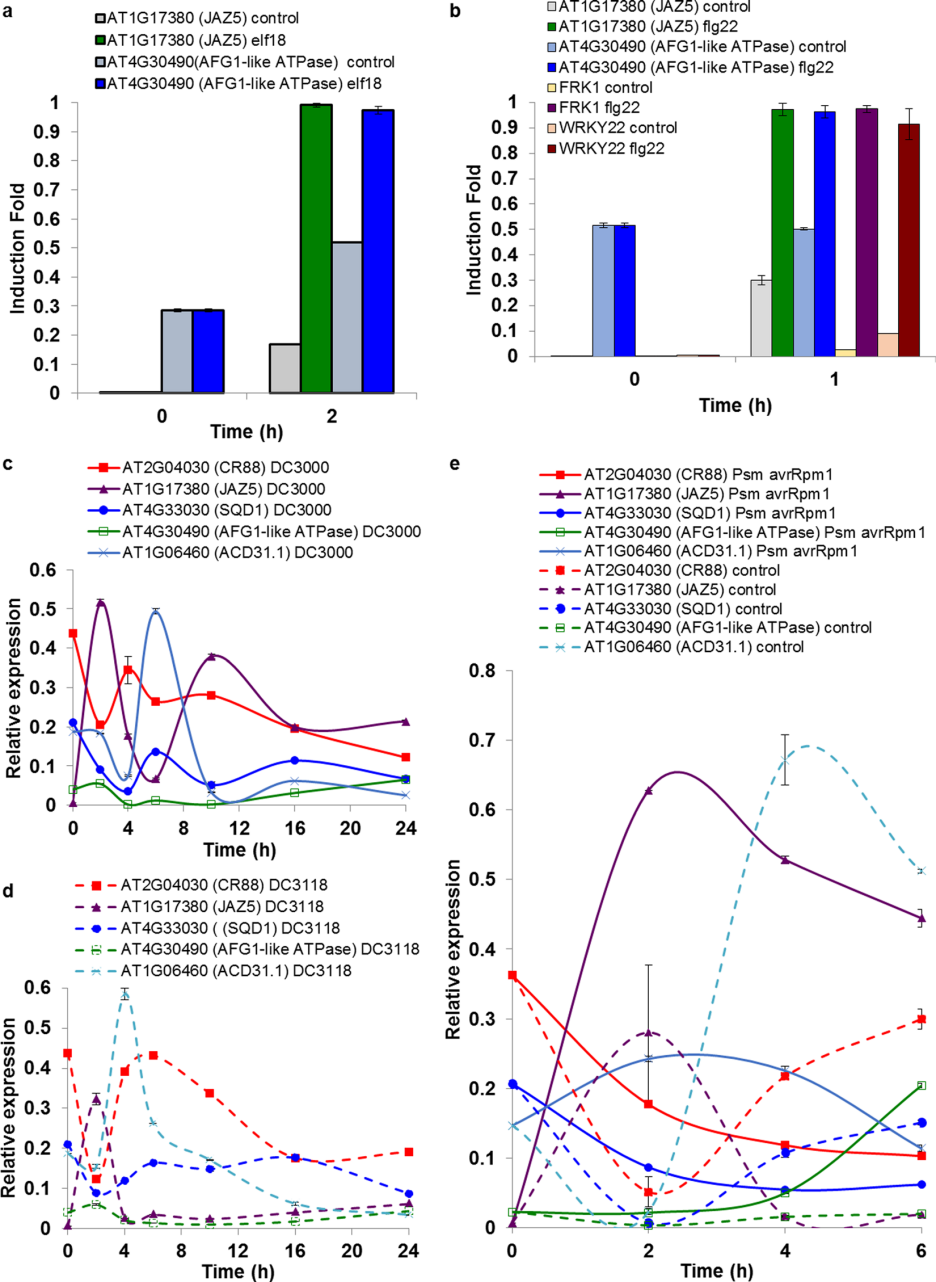
qRT-PCR analyses of five selected genes upon treatments with diverse stimuli and bacterial pathogens. Accumulation transcripts of the indicated genes is revealed upon treatments with elf18 or control (**a**), flg22 (**b**), DC3000 (**c**), DC3118 (**d**) and *Pseudomonas syringae* expressing AvrRpm1 (**e**). Data represent the mean and standard error of two technical replicates.

To demonstrate the roles of these selected genes in MTI and ETS, we performed a time-course qRT-PCR (quantitative Reverse Transcription-Polymerase Chain Reaction) on RNA extracted from wild-type leaf tissue treated with two MAMPs: flg22 and elf18, DC3000, DC3118 (a DC3000 mutant strain that lacks the ability to deliver phytotoxin coronatine, COR), and strain expressing AvRpm1 (an avirulent strain that triggers ETI). Upon elf18 treatment, we observed 100 fold and three fold transcript induction for At1g17380 (JAZ5) and At4g30490 (AFG1-like ATPase), respectively, while we did not notice any significant change in the mRNA levels for the remaining three genes (Fig. 4a). Similar results were obtained from the qRT-PCR analyses pertaining to flg22 treatments (Fig. 4b). Two very well established MAMPs-related molecular markers, *FRK1* (flg22-receptor kinase 1) and *WRKY22* that are induced rapidly in response to flg22 and other molecular patterns^63^, were used as positive controls (Fig. 4b). Subsequently, we monitored the expression patterns of these five genes upon infection with DC3000 and DC3118 over the course of 24 hours. We detected biphasic induction of At1g17380 (JAZ5) in response to DC3000 but not DC3118 (Fig. 4c and d), which aligns with a recent report indicating the roles of JAZ5 in basal defense and secondary plant immune responses^51^. Similarly, we also revealed the activation of At2g04030 (CR88), At1g06460 (ACD31.1) and At4g33030 (SQD1) genes at early and late phases of infection with different amplitude suggesting their roles in the onset of ETS (Fig. 4c and d). Finally, we demonstrated a moderate induction of At4g30490 (AFG1-like ATPase) in the very late phase of DC3000 and DC3118 treatments (Fig. 4c and d).

To understand if ETI affects the transcription of those five genes, we quantified the transcript levels in response to an avirulent pathogen expressing AvrRpm1. As shown in Fig. 4e, the transcript accumulation of At1g17380 *(JAZ5)* and At3g30490 (AFG1-like ATPase) was highly induced during ETI and this induction persisted throughout the tested period. The transcript of At1g06460 (ACD31.1) was induced by avirulent pathogen at 2 h post infection; however, the expression pattern was reversed at 4 h and 6 h post infection (Fig. 4e). Likewise, At2g04030 (CR88), and At4g33030 (SQD1) follow a similar trajectory of expression (Fig. 4e).

**Figure 5:**
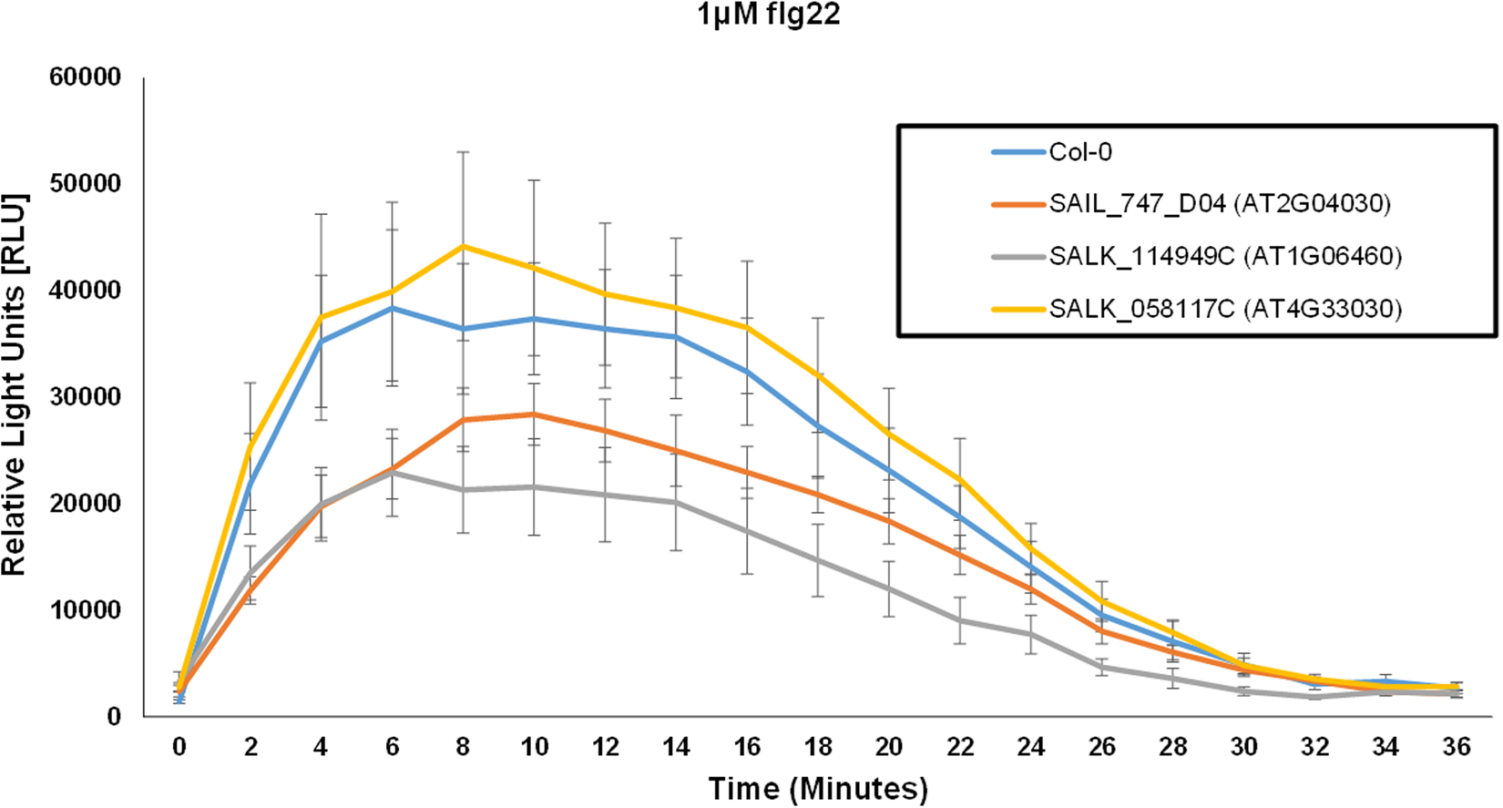
Reactive oxygen species (ROS) burst in *Arabidopsis* leaves triggered by flg22. ROS profile of Col-0, SAIL_747_D04 (AT2G04030), SALK_114949 C (AT1G06460) and SALK_058117 C (AT4G33030) at the indicated time points are shown. Leaf samples were harvested from four-week-old *Arabidopsis* plants and ROS burst was measured by a luminol-based assay immediately after addition of 1 μM flg22. The data are shown as means ± SEs (standard error) from 8 leaf discs.

**Figure 6:**
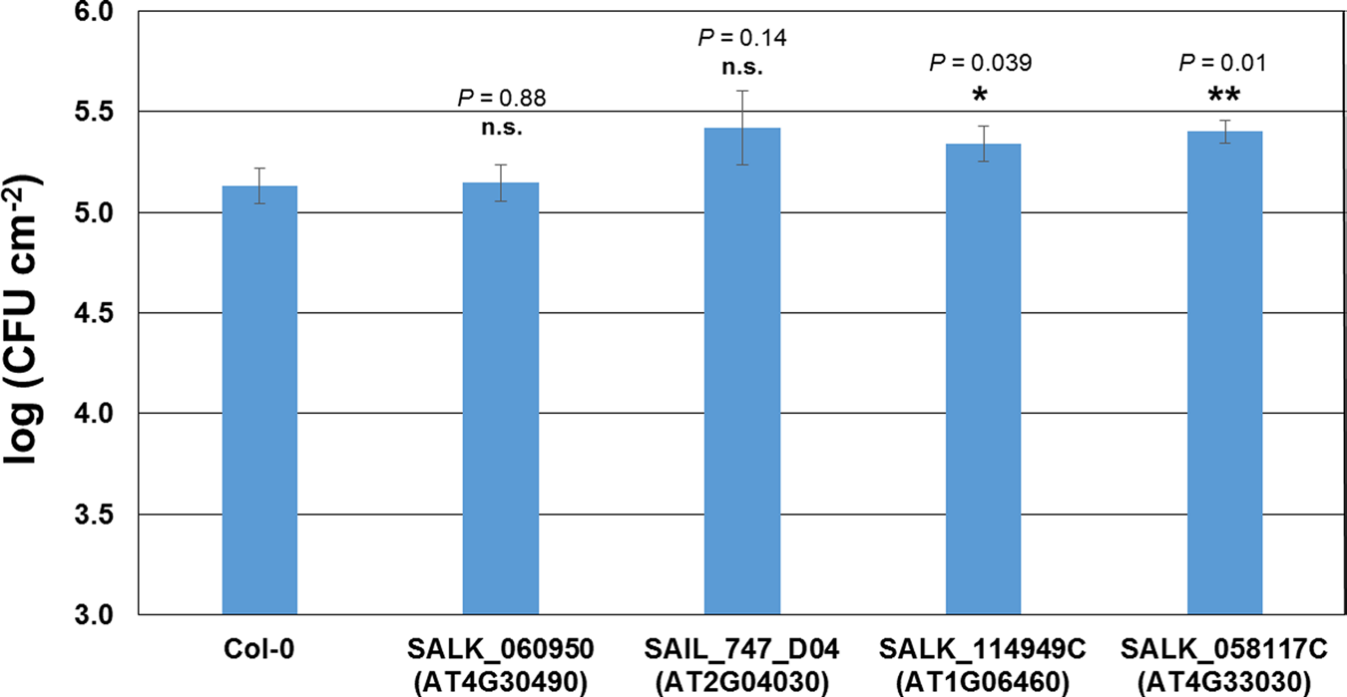
At2g04030 (CR88) and At1g06460 (ACD31.1) displayed increased susceptibility to DC3000*hrcC* ^−^. Growth of DC3000*hrcC* ^−^ in plants were quantified three days post infiltration (OD_600 nm_ = 0.0002). Results presented are average ± stand error. *n* = 5, *p < 0.05, asterisks indicate the difference is significant compared to Col-0 by two-tailed Student’s *t-*test.

Our network and extensive qRT-PCR analyses indicate the potential roles of these five genes in plant immune system. Indeed, the overaccumulation of *JAZ5* mRNA, which coincides with the overproduction of JA and occurs at the late phase of infection with DC3000, has been recently shown^51^. To elucidate the potential functions of the remaining four genes (*CR88*, *SQD1*, *AFG1-like ATPase* and *ACD31.2*) in plant immunity, we obtained their loss-of-function mutants. Diminished or complete loss of transcripts was detected in the respective mutants compared to Col-0 (Supplementary Fig. S3). To examine the involvement of these four genes in plant immunity, we observed reactive oxygen species (ROS) production triggered by flg22 treatment over the course of 36 minutes. A rapid and transient production of ROS burst is another hallmark of early MTI^63^ that allows mounting effective defenses against the invading biotrophic pathogens, including DC3000. We demonstrated that mutants corresponding to At2g04030 (CR88) and At1g06460 (ACD31.1) exhibited less pronounced ROS production compared to Col-0 (Fig. 5 and Supplementary Fig. S4). To provide the direct evidence of the involvement of At4g30490 (AFG1-like ATPase), At2g04030 (CR88), At1g06460 (ACD31.1) and At4g33030 (SQD1), in MTI, we infected their mutant plants with DC3000*hrcC* ^−^^64^ (a type III secretion system deficient DC3000 mutant strain). We showed that plants lacking functional At2g04030 (CR88) and At1g06460 (ACD31.1) displayed increased susceptibility to DC3000*hrcC* ^−^, which corroborates our ROS burst data (Fig. 6). While the underlying mechanisms by which these genes participate in plant immune responses are focal points of future investigations, here we showed how systems level analyses could lead to discover novel players in plant disease resistance.

## Discussion

This study highlights the importance of systems biology approaches that utilize integrative experimental data from diverse sources, mathematical modeling, and computational tools to discover novel components of a complex system^1, 2, 6, 10^. Lending further support to the value and importance of topology analyses in different networks, we discovered that the effector targets are closer to DEGs pairs (Fig. 1). Likewise, the shortest paths in combination with other centrality measures revealed novel genes, pathways, and microbial communities in other biological systems^44, 45, 65–67^. For instance, network topology analyses, *i.e*. shortest path on a large-scale dataset led to the discovery of a new signaling axes-related to Syk, a protein tyrosine kinase, which negatively affects tumor development and progression^66^. Similarly, network analysis in human-*Burkholderia mallei* recently showed that pathogen virulence factors interact with multifunctional host hub proteins to simultaneously manipulate a wide range of host cellular processes^65^. In another study, viral proteins also interact with key host proteins to hijack diverse signaling pathways for infectivity^44, 45^. Shortest path centrality successfully identified human proteins targeted by hepatitis C virus as well as influenza A/H7N9 virus^44, 45^. Given that effector targets are highly connected proteins and pairs of immune interactors are closer to each other than non immune interactors in AI-1_MAIN_^30, 32, 33^, we concluded that effector targets relay biological information using DEGs in Arabidopsis.

Integration of our diverse “-omics” data including transcriptome and interactome revealed unique features of effector targets. Temporal dynamic relationships of DEGs with effector targets in conjunction with protein-protein interactions led us to discover that effector targets are sequentially expressed over the course of infection with virulent or MTI-inducing bacterial strains (Figs 2 and 3). Since single bacterial effector can target multiple host proteins as well as multiple effectors from the same bacterial strain can directly target a common set of host proteins, we hypothesized that the sequential regulation of effector targets may coincide with the chronological delivery of bacterial effectors into the host cells. Moreover, this may also explain the functional redundancy of the bacterial effectors in modulating MTI. It is also a significant discovery in furthering our knowledge in Arabidopsis innate immunity that led us to determine how MTI signals are transmitted through utilizing direct targets of effectors (primary effector targets) and 2° targets. Overall, our network biology-based analyses shed light on how defense-inducing and disease causing bacterial strains sequentially and temporally regulate these high value targets to establish innate immunity or disease susceptibility, respectively.

MTI- and ETS-regulated unique and shared effector targets provided additional insights into the regulatory mechanisms of the activation of innate immunity and establishment of disease resistance. Specifically, our Gene Ontology (GO) enrichment analysis for these DC3000*hrpA* ^−^ regulated effector targets and 2° targets showed high enrichment for the GO terms “transmembrane”, “chloroplast”, “regulation of transcription”, “endoplasmic reticulum (ER)”, and “ATP binding” (Supplementary Fig. S5a,b and Supplementary Table S6). These data indicate that plants transmit MTI signals by utilizing innate immune players such as membrane and ER-related peptides as well as ATP binding proteins. Since fitness costs associated with plant immune responses have been previously demonstrated^68–70^, the GO term “chloroplast” relates to growth-defense tradeoffs during MTI. Importantly, we discovered that the amplitude of over 71% shared targets of MTI and ETS is altered (Fig. 3 and Supplementary Fig. S2) that coincides with the delivery of bacterial effectors (Supplementary movie S2). In contrast, GO enrichment analyses for DC3000-regulated effector unique targets revealed two prominent categories of genes, *i.e*. “metabolic pathway (amino acid, purines and secondary metabolites)” and “zinc binding” that appeared to be distinctive to DC3000 compared to DC3000*hrpA* ^−^ regulated genes (Supplementary Fig. S5c,d and Supplementary Table S6). While the significance of metabolic pathways in plant immunity has been previously demonstrated^56^, the catabolism of carbohydrates, amino acids, and lipids in plant nutritional immunity is an emerging concept and requires future investigations. Indeed, our dynamic complexes contain genes encoding several metabolic-related proteins such as SQD1, putative pyruvate kinase and HCF244 (Supplementary Tables S4 and S5). Likewise, the roles of transition metals including zinc in plant-bacterial interactions remain to be fully understood^71^, whereas the importance of zinc in human-pathogen interactions, especially in nutritional immunity and ROS production and activation of immunological signals has been well illustrated^72^. Overall, our integrative analyses followed by isolation of dynamic complexes discovered metabolic pathways and transition metal genes that may be targeted by DC3000 to overcome MTI and establish ETS.

With the availability of large-scale transcriptome and interactome data sets, the use of network-based approaches is emerging to identify structural and functional modules as well as essential and most influential nodes within a network^1, 5, 6, 10, 38, 73, 74^. Recently, a comparative network analysis, which combined transcriptional and PPI datasets, discovered common as well as contrasting sub-networks for biotrophic and necrotrophic pathogens^75^. Importantly, another study utilized the same set of transcriptomic data generated by Lewis *et al*.^29^ and performed a differential co-expression analysis^76^. This led to discover that differentially co-expressed genes play crucial roles in plant immunity^76^. In our study, we detected the existence of dynamic protein complexes at various phases of bacterial infection during MTI and ETS. We further inferred the plant immune functions for five dynamic complexes containing genes that are regulated by DC3000 and/or DC3000hrp*A* ^−^ strains. In addition, we characterized the functions of a set of these novel components in plant immunity and demonstrated the requirement of at least three players in MTI (Figs 4, 5 and 6).

Although the underpinning molecular mechanisms remain to be explored, our study provides the prospect of several new components that can be investigated in conjunction with plant immunity. While this study formed the basis for isolating dynamic protein-protein interaction complexes *in silico*, future research will entail genetics, biochemical and pathology experiments to decipher the individual and combinatorial roles of these novel players in plant immunity. This integrative “-omics” platform could potentially be applicable to other plant-pathogen interactions networks to identify dynamic protein complexes, while fostering broad-spectrum disease resistance strategies through genetic engineering in agriculturally important crop plants.

## Materials and Methods

### Data sources, bioinformatics software, and network analyses

The principal foundation of this study is a static PPI network, AI-1_MAIN_ and effector targets reported in PPIN-1 as well as PPIN-2^30, 32, 33^. Interactome data pertinent to AI-1_MAIN_, PPIN-1, PPIN-2 as well as Nucleic Acid-Programmable Protein Array (NAPPA) can be downloaded from the following sites.

http://signal.salk.edu/interactome/index2.html

http://interactome.dfci.harvard.edu/A_thaliana/index.php?page = home

http://interactome.dfci.harvard.edu/A_thaliana/index.php?page = display.

Raw temporal gene expression datasets (GSE56094) and differentially expressed genes for DC3000*hrpA* ^−^ and DC3000 were downloaded from GEO Omnibus and Lewis *et al*.^29, 77^. We performed DEGs analysis as described by Lewis *et al*.^29^. This yields a total of 9,782 and 7,119 differentially expressed genes (DEGs) for DC3000*hrpA* ^−^ and DC3000, respectively. The AI-1_MAIN_ network were subjected to Python-based NetworkX^78^ analyses. Specifically, we computed information centrality and shortest paths with default parameters (unweighted graphs *i.e*. all edges are treated the same). Information centrality is an alternative of closeness centrality based on actual resistance among nodes in a given network and determines the flow of information from one to other node based on length of subnetworks of a network^79^. The information centrality *IC*(*i*) of node *i* in a graph G was calculated as:

$$C(i)={\lceil \frac{1}{n}\sum _{j}\frac{1}{{I}_{ij}}\rceil }^{-1}$$

where, n is the total number of nodes and $\,{I}_{ij}=({r}_{ii}+{r}_{jj}-{r}_{ij})-1$, ${r}_{ij}$ is an element of R matrix. D is a diagonal matrix of each node with weighted degree and J is a matrix with all its elements are 1. Then, $R=\,({r}_{ij})=[D-A+J]-1$. Computationally, ${I}_{ii}\,$is defined as infinite. Hence, $\frac{1}{{I}_{ij}}=0.$

The shortest paths between an effector and DEGs in AI-1_MAIN_ were calculated. The total numeral of shortest paths traverse through a node $\,(v)$, is calculated as

$$g(v)=\sum _{s\ne v\ne t}\frac{{\sigma }_{st}\,(v)}{{\sigma }_{st}}$$

where, ${\sigma }_{st}$is the sum of shortest paths from source node “s” to target node “t” and ${\sigma }_{st}(v)$ is the total number of paths that traverse through node $(v)$.

Network analyses and visualization were performed using Cytoscape^80–82^. Clustering calculations and interactive heatmaps of effector targets were generated by custom made scripts using heatmaply v0.7.0 package for R language. Integration of protein-protein pairs was performed by in-house Python and Java scripts.

### Time-Specific interactive dynamic PPI network and construction of dynamic subnetworks

Time-specific DEGs were filtered based on GP2S algorithm as descried in Lewis *et al*.^29^. We set a cut-off of Log2 +1 and −1 for up- and down-regulation and extracted a set of DEGs for each time point for DC3000*hrpA* ^−^ and DC3000 for dynamic PPI network and dynamic subnetwork analyses. These time-specific up- and down-regulated genes were utilized as “node attribute” to visualize dynamic AI-1_MAIN_ using Cytoscape^80–82^. The extracted DEGs at each time point and static PPI network, AI-1_MAIN_ were utilized to identify 13 subnetworks (set of protein-protein pairs as previously described in Wang *et al*.^83^), one for each time in DC3000*hrpA* ^−^ and DC3000 using in-house Java script,. These time specific protein-protein pairs are termed as dynamic PPI subnetworks. Dynamicity of AI-1_MAIN_ and subnetworks associated with DC3000*hrpA* ^−^ and DC3000 was displayed by generating GIFs using individual snapshots for each time point.

### Effector targets or 1^st^ degree (1°) effector targets identification

Effector targets for each time point in DC3000*hrpA* ^−^ and DC3000 and were identified. A total of 104 effector targets associated with DC3000*hrpA* ^−^ and DC3000 were found to be DEGs. 45 effector targets were shared between DC3000*hrpA* ^−^ and DC3000, while 41 and 18 DEGs were unique to DC3000*hrpA* ^−^ and DC3000, respectively. Hierarchical clustering based on Euclidian distance was computed and heat-maps were generated for the expression values of effector targets in DC3000*hrpA* ^−^ and DC3000 by heatmaply package of R language.

### 2^nd^ degree (2°) effector targets identification

We subjected 104 differentially expressed 1° effector targets to 5,664 edges of AI-1_MAIN_ to identify the interacting partners of effector targets (2°effector targets) that are also DEGs either in DC3000*hrpA* ^−^ and DC3000. These extracted 1° and 2° effector target nodes were used as “node attribute” to visualize dynamic AI-1_MAIN_ using Cytoscape^80–82^. Gene Ontology (GO) enrichment analysis on genes corresponding to effector targets and 2° effector targets was done using DAVID Functional Annotation Bioinformatics Microarray Analysis^84^.

### Plant material and growth conditions

Arabidopsis (*Arabidopsis thaliana*) Col-0 was used as the wild-type control in all experiments. T-DNA insertion lines for AT2G04030 (SAIL_747_D04), AT1G06460 (SALK_114949 C), AT4G33030 (SALK_058117 C), and AT4G30490 (SALK_060950) were obtained from Arabidopsis Biological Resource Center (ABRC). T-DNA and gene specific primers (Supplementary Table S7) were designed using SIGnAL’s T-DNA primer website http://signal.salk.edu/tdnaprimers.2.html. Homozygosity of these mutants was confirmed using genotyping PCR using Phire Plant Direct PCR Kit (Thermo Fisher). The lack of accumulation of transcripts was further confirmed by qRT-PCR analysis using respective primers (Supplementary Table S7). Col-0 and mutant plants used in this study were grown at 21 °C with a 12/12 hours light/dark photoperiod in environmentally controlled chambers.

### Stress treatments, RNA isolation and quantitative Real Time PCR (qRT-PCR)

4-week old Arabidopsis plants were syringe-infiltrated with 10 μM flg22 (Genscript) or 10 μM elf18 (Genscript) and samples were collected at designated time points. To induce effector-triggered immunity, 4-week old plants were syringe-infiltrated with *P. syringae* pv. *maculicola* ES4326/AvrRpm1 (OD_600 nm_ = 0.1) and samples were collected at designated time points. 4-week old plants were spray inoculated with DC3000 (OD_600 nm_ = 0.2, 0.02% Silwet L-77) or DC3118 (OD_600 nm_ = 0.2, 0.02% Silwet L-77) and samples were collected at designated time points.

Total RNA was extracted from collected samples using RiboZol (AMRESCO) and genomic DNA contamination was removed using DNase I (Ambion). mRNA was converted into cDNA using the SuperScript III first-strand RT-PCR kit (Invitrogen). Gene expression analysis was conducted with GoTaq qPCR Master Mix (Promega) using gene-specific primers in a RealPlex S MasterCycler (Eppendorf). Primers used for this study are listed in the Supplementary Table S7.

### Measurement of reactive oxygen species (ROS) burst and pathogen assay

Oxidative burst in Arabidopsis leaves was detected using a luminol-based assay as described previously by Macho *et al*.^85^. Briefly, 4-mm leaf discs from 4-week-old Arabidopsis plants were incubated in 100 μl sterile distilled water overnight at room temperature. Immediately before treatment, 100 μl water was replaced with equal amount of luminol/peroxidase working solution containing 1 μM flg22 (Sigma), 34 μg/ml luminol (Sigma) and 20 μg/ml horseradish peroxidase (Sigma). Luminescence was measured on a 96-well plate reader immediately after treatment and recorded every 2 min until 36 min. Four-week-old plants were syringe-infiltrated with DC3000*hrcC* ^*−*^ bacterial solution OD_600 nm_ = 0.0002 in 10 mM MgCl_2._ Four leaves per plant and five plants per genotype were used for pathogen quantification through serial dilution.

### Statistical analyses

R language was used to perform Student’s *t*-test (distribution of information centrality and pathology assay) and Chi-Square Test of Independence (shortest paths). Average, mean, standard deviations and standard error were calculated using Microsoft Excel 2016 (insert function). Mean and standard error were performed on two technical replications for qRT-PCR analyses. ROS burst data are shown as means and ± standard error (SEs) from 8 leaf discs. Moreover, means and ± standard error (SEs) were performed on five independent biological replications for pathology assay.

### Data Availability

All data generated or analyzed during this study are included in this published article (and its Supplementary Information files). Mutants corresponding to four genes in the current study are available from the corresponding author on reasonable request.

## Electronic supplementary material

Supplementary information

Supplementary Table S1

Supplementary Table S2

Supplementary Table S3

Supplementary Table S4

Supplementary Table S5

Supplementary Table S6

Supplementary Table S7

Supplementary movie S1 online

Supplementary movie S2 online
