# Supplementary material for: Global temporal dynamic landscape of pathogen-mediated subversion of Arabidopsis innate immunity
Source: Sci Rep. 2017 Aug 10;7:7849. doi: 10.1038/s41598-017-08073-z (PMC5552879; doi:10.1038/s41598-017-08073-z)
Supplement: Supplementary file 1 — Supplementary information [file 41598_2017_8073_MOESM1_ESM.doc]

**Title**

Global temporal dynamic landscape of pathogen-mediated subversion of Arabidopsis innate immunity

Bharat Mishra^1^, Yali Sun^1^, Hadia Ahmed^2^, Xiaoyu Liu^1^ & M. Shahid Mukhtar^1,3^*

## Affiliations

^1^ Department of Biology, University of Alabama at Birmingham

^2^ Department of Computer & Information Sciences, University of Alabama at Birmingham

^3^ Nutrition Obesity Research Center, University of Alabama at Birmingham

***Correspondence to (**[**smukhtar@uab.edu**](mailto:smukhtar@uab.edu)**)**

**Supplementary Information**

**Supplementary Figure Legends**

Supplementary Figure S1. Measurement of shortest path between effector targets and DEGs, and non effector targets and DEGs. 11,070 cumulative DEGs for DC3000*hrpA^-^* and DC3000 treatments were extracted for this experiment (Chi-Square Test of Independence *P* < 2.2 x 10^-16^).

Supplementary Figure S2: The heat map of shared effector targets between DC3000*hrpA^-^* and DC3000. Hierarchical clustering is performed based on Euclidean distance for DC3000*hrpA^-^*- (left) and DC3000-regulated (right) genes. Heat maps of up-regulated genes (**a**) and down-regulated (**b**) in orange and blue colors, respectively at the indicated time points are shown.

Supplementary Figure S3. qRT-PCR analysis to measure the transcript levels in the mutants corresponding to *AT2G04030* (**a**)*, AT1G06460* (**b**)*, AT4G33030* (**c**), and *AT4G30490* (**d**) genes.

Supplementary Figure S4. Reactive oxygen species (ROS) burst in the leaves of Col-0 and SALK_060950 plants triggered by flg22. The data are shown as means ± SEs from 8 leaf discs.

Supplementary Figure S5. DC3000*hrpA^-^*- and DC3000-regulated unique effector targets. Heat map depiction of unique effector targets that are up- (**a** and **c**) and down-regulated (**b** and **d**) by DC3000*hrpA^-^*- (**a** and **b**) and DC3000 (**c** and **d**) treatments. Unique Effector targets in DC3000*hrpA^-^*and DC3000 clustered based on Euclidean distance.

**Supplementary Tables Legends**

Supplementary Table S1. DC3000*hrpA^-^*- and DC3000-regulated differentially expressed genes (DEGs) and effector targets.

Supplementary Table S2. First significant divergence in the expression gradient for effector targets.

Supplementary Table S3. List of first degree and second degree effector targets.

Supplementary Table S4. DC3000*hrpA^-^*- and DC3000-related dynamic complexes.

Supplementary Table S5. Gene description and dynamic expression status in DC3000*hrpA^-^*- and DC3000 complexes of five selected genes.

Supplementary Table S6. Significantly enriched GO terms in the effector targets and degree effector targets.

Supplementary Table S7. Primers used to perform genotyping and qRT-PCR.

**Supplementary Movies Legends**

Supplementary movie S1. DC3000*hrpA^-^*-mediated dynamic regulation of genes corresponding to effector targets and their direct and indirect interactors in AI-_MAIN_. Non DEGs are removed from AI-_MAIN_ for clarity.

Supplementary movie S2. DC3000-mediated dynamic regulation of genes corresponding to effector targets and their direct and indirect interactors in AI-_MAIN_. Non DEGs are removed from AI-_MAIN_ for clarity.


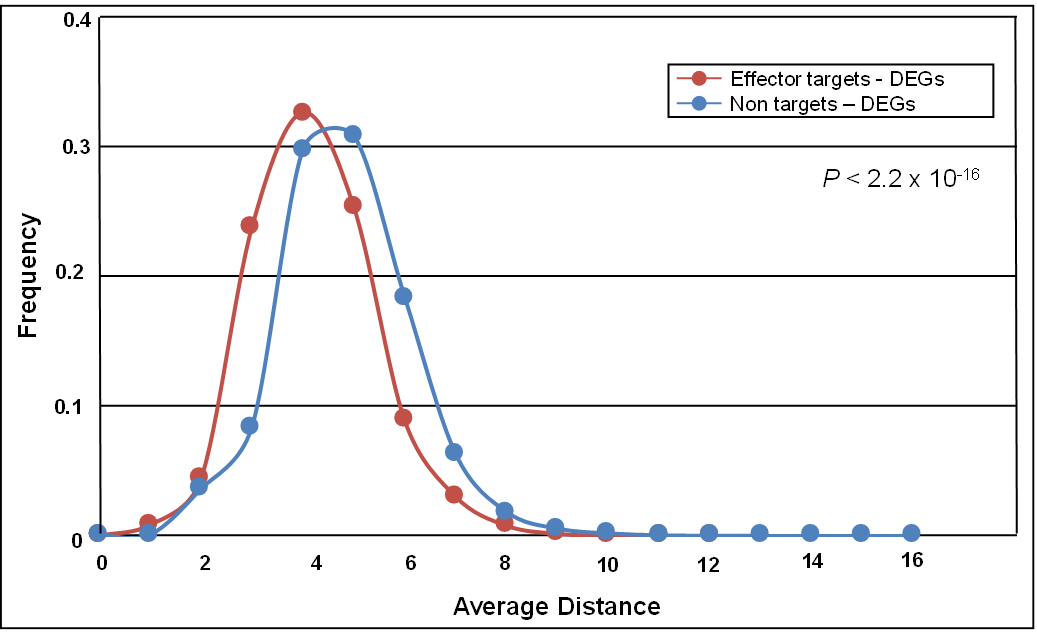


Supplementary Figure S1. Measurement of shortest path between effector targets and DEGs, and non effector targets and DEGs. 11,070 cumulative DEGs for DC3000*hrpA^-^* and DC3000 treatments were extracted for this experiment (Chi-Square Test of Independence *P* < 2.2 x 10^-16^).


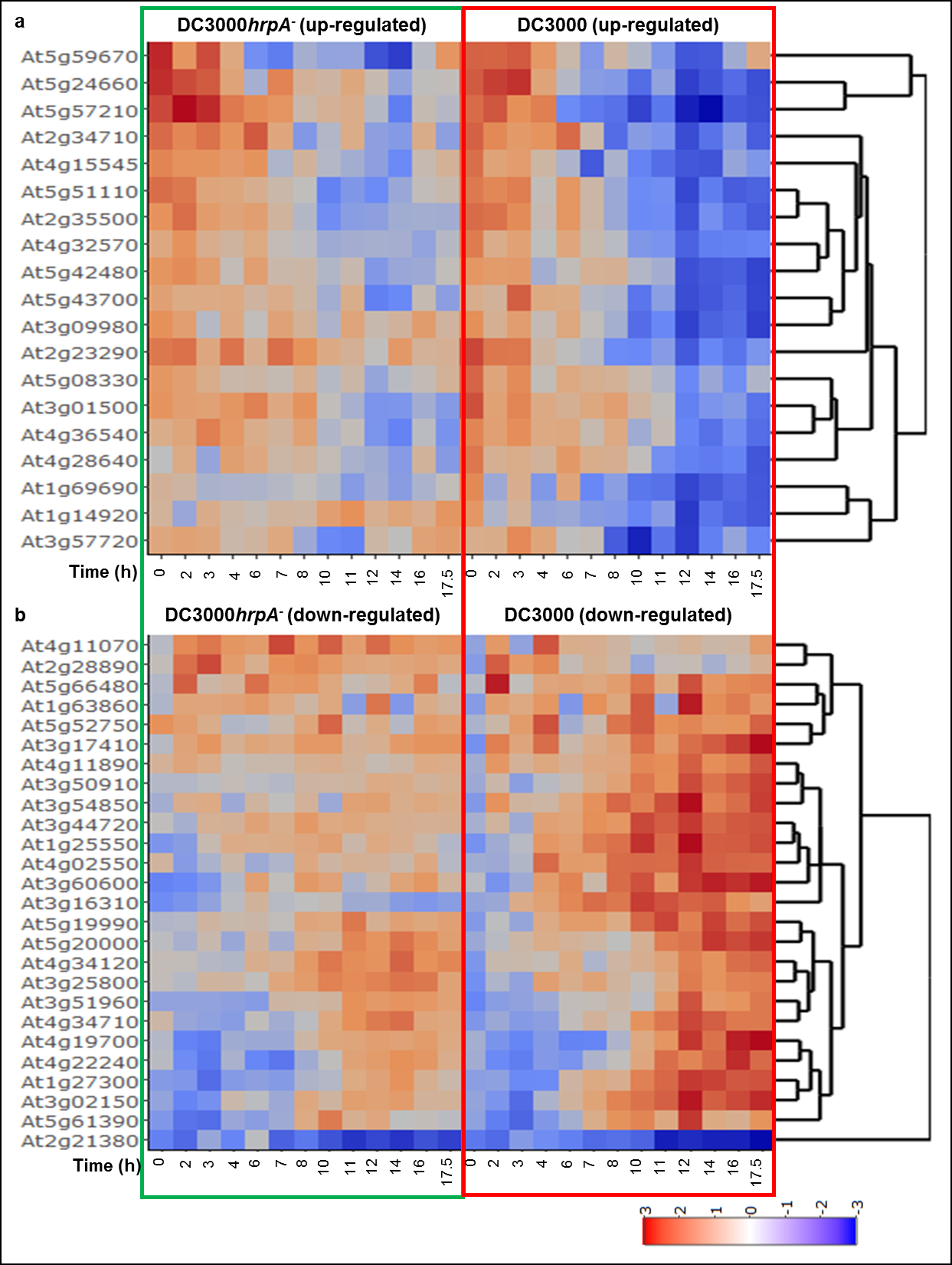


Supplementary Figure S2: The heat map of shared effector targets between DC3000*hrpA^-^* and DC3000. Hierarchical clustering is performed based on Euclidean distance for DC3000*hrpA^-^*- (left) and DC3000-regulated (right) genes. Heat maps of up-regulated genes (**a**) and down-regulated (**b**) in orange and blue colors, respectively at the indicated time points are shown.


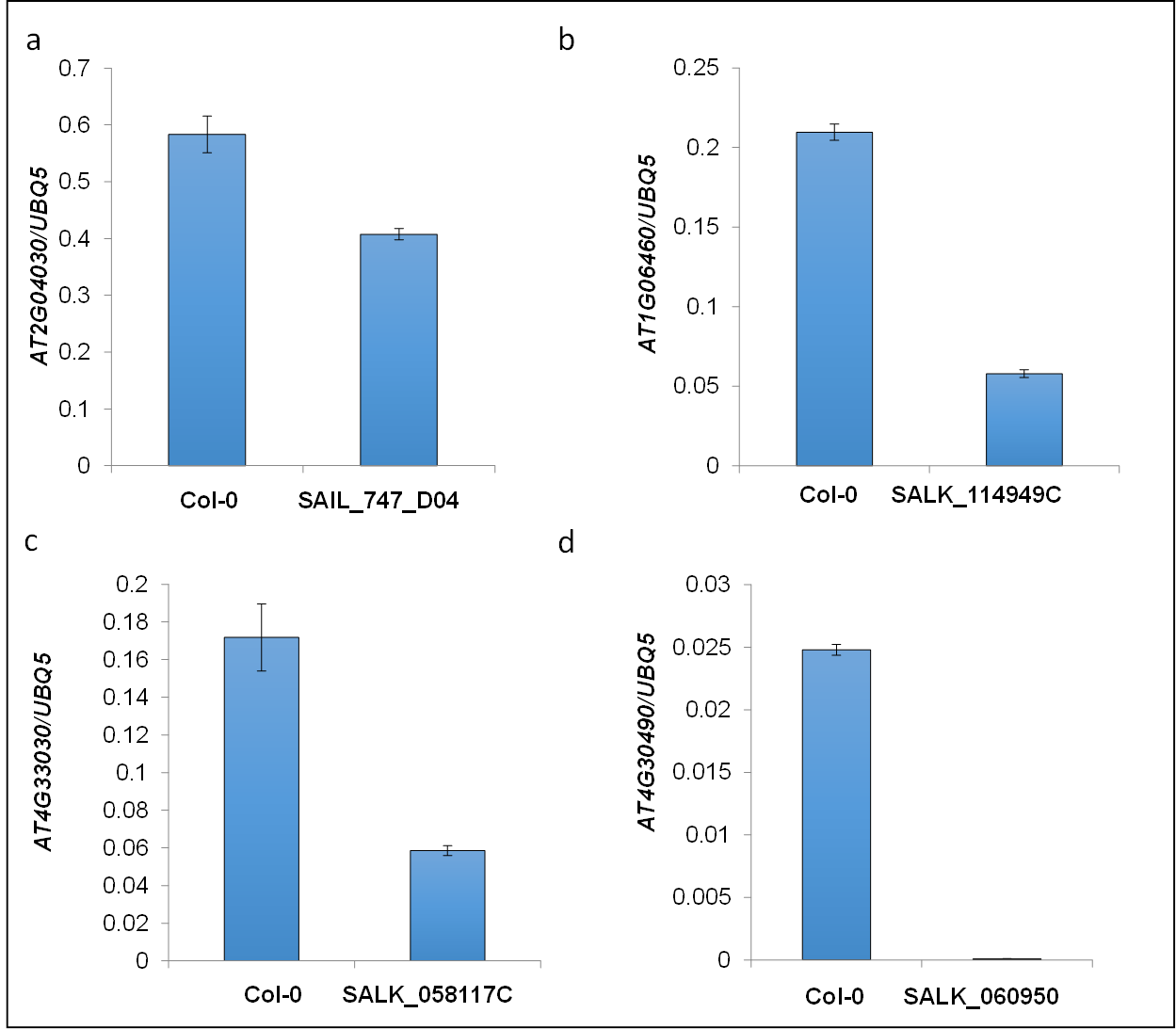


Supplementary Figure S3. qRT-PCR analysis to measure the transcript levels in the mutants corresponding to *AT2G04030* (**a**)*, AT1G06460* (**b**)*, AT4G33030* (**c**), and *AT4G30490* (**d**) genes.


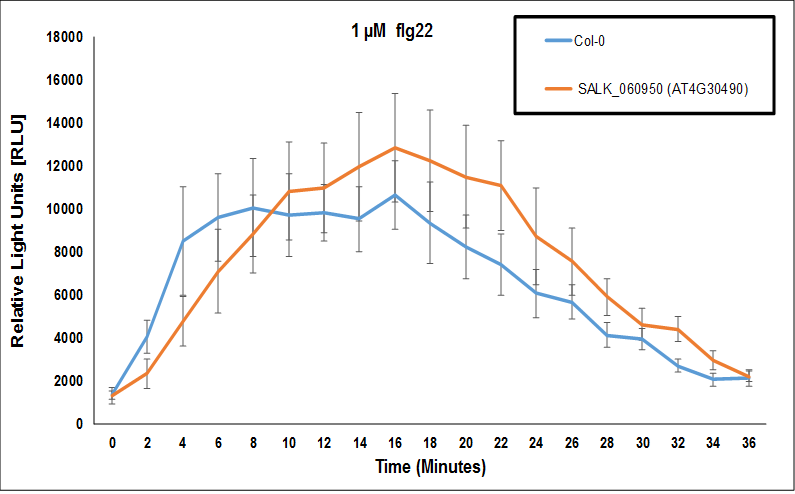


Supplementary Figure S4. Reactive oxygen species (ROS) burst in the leaves of Col-0 and SALK_060950 plants triggered by flg22. The data are shown as means ± SEs from 8 leaf discs.


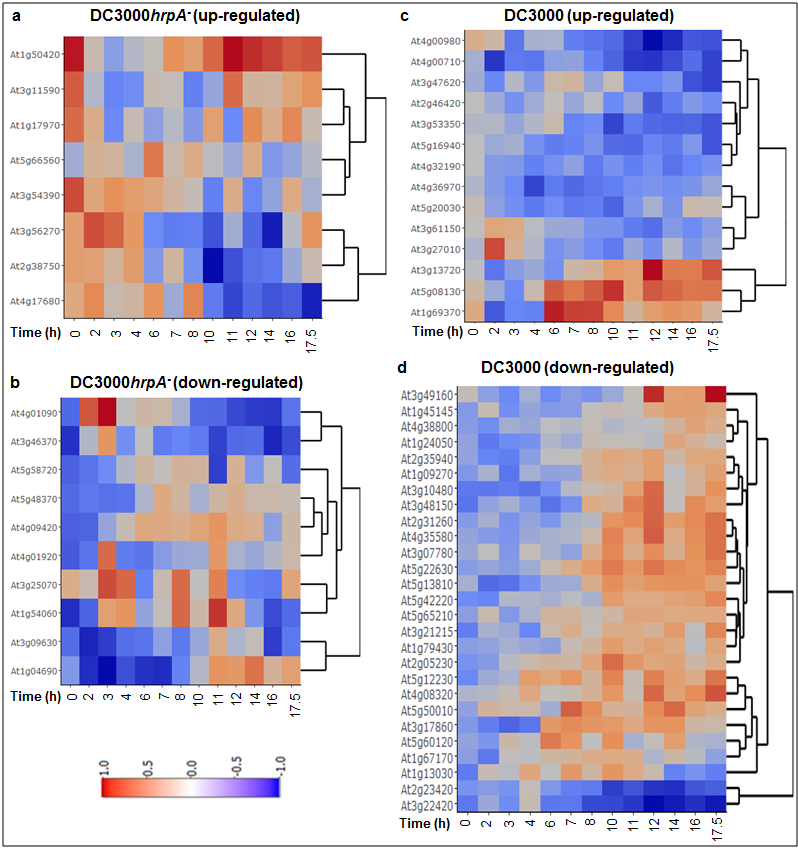


Supplementary Figure S5. DC3000*hrpA^-^*- and DC3000-regulated unique effector targets. Heat map depiction of unique effector targets that are up- (**a** and **c**) and down-regulated (**b** and **d**) by DC3000*hrpA^-^*- (**a** and **b**) and DC3000 (**c** and **d**) treatments. Unique Effector targets in DC3000*hrpA^-^*and DC3000 clustered based on Euclidean distance.
