# Supplementary figures and images for: Global temporal dynamic landscape of pathogen-mediated subversion of Arabidopsis innate immunity
Source: Sci Rep. 2017 Aug 10;7:7849. doi: 10.1038/s41598-017-08073-z (PMC5552879; doi:10.1038/s41598-017-08073-z)

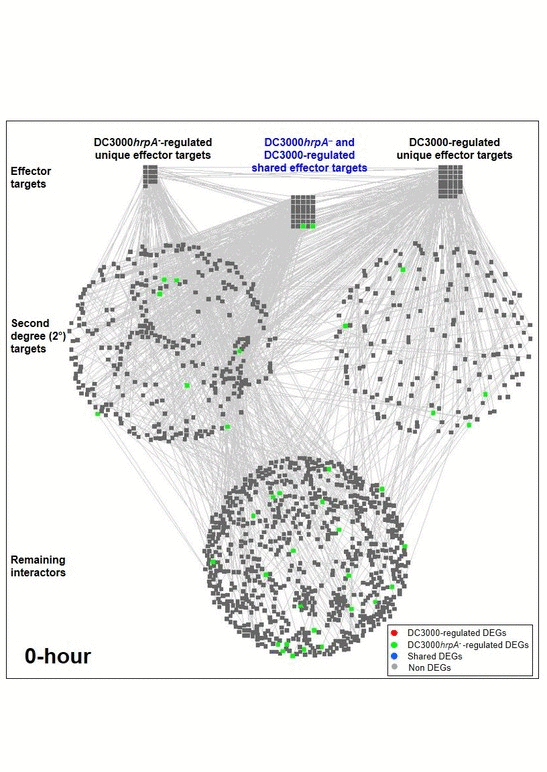

Supplement: Supplementary file 9 — Supplementary movie S1 online [file 41598_2017_8073_MOESM9_ESM.gif]

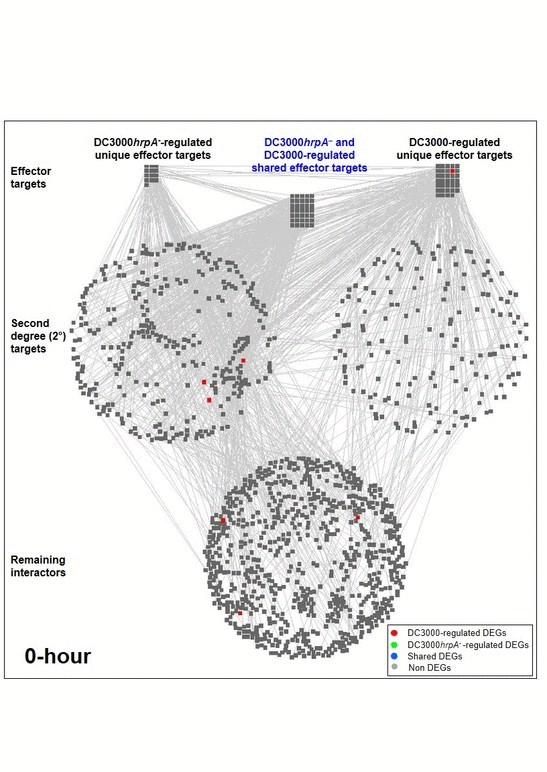

Supplement: Supplementary file 10 — Supplementary movie S2 online [file 41598_2017_8073_MOESM10_ESM.gif]
